# A potential therapeutic target in traditional Chinese medicine for ulcerative colitis: Macrophage polarization

**DOI:** 10.3389/fphar.2022.999179

**Published:** 2022-09-06

**Authors:** Zhihua Yang, Shanshan Lin, Wanying Feng, Yangxi Liu, Zhihui Song, Guiyun Pan, Yuhang Zhang, Xiangdong Dai, Xinya Ding, Lu Chen, Yi Wang

**Affiliations:** ^1^ Institute of Traditional Chinese Medicine, Tianjin University of Traditional Chinese Medicine, Tianjin, China; ^2^ State Key Laboratory of Component-Based Chinese Medicine, Tianjin University of Traditional Chinese Medicine, Tianjin, China; ^3^ National Clinical Research Center for Chinese Medicine Acupuncture and Moxibustion, First Teaching Hospital of Tianjin University of Traditional Chinese Medicine, Tianjin, China

**Keywords:** macrophage polarization, traditional Chinese medicine, ulcerative colitis, potential mechanisms, gut microbiota, signaling pathway

## Abstract

Intestinal macrophages are the main participants of intestinal immune homeostasis and intestinal inflammation. Under different environmental stimuli, intestinal macrophages can be polarized into classical activated pro-inflammatory phenotype (M1) and alternative activated anti-inflammatory phenotype (M2). Its different polarization state is the “guide” to promoting the development and regression of inflammation. Under normal circumstances, intestinal macrophages can protect the intestine from inflammatory damage. However, under the influence of some genetic and environmental factors, the polarization imbalance of intestinal M1/M2 macrophages will lead to the imbalance in the regulation of intestinal inflammation and transform the physiological inflammatory response into pathological intestinal injury. In UC patients, the disorder of intestinal inflammation is closely related to the imbalance of intestinal M1/M2 macrophage polarization. Therefore, restoring the balance of M1/M2 macrophage polarization may be a potentially valuable therapeutic strategy for UC. Evidence has shown that traditional Chinese medicine (TCM) has positive therapeutic effects on UC by restoring the balance of M1/M2 macrophage polarization. This review summarizes the clinical evidence of TCM for UC, the vital role of macrophage polarization in the pathophysiology of UC, and the potential mechanism of TCM regulating macrophage polarization in the treatment of UC. We hope this review may provide some new enlightenment for the clinical treatment, fundamental research, and research and development of new Chinese medicine of UC.

## 1 Introduction

Ulcerative colitis (UC) is a chronic inflammatory bowel disease with persistent damage of colonic epithelial mucosa as the main pathological change (Schlegel et al., 2021), the clinical manifestations are persistent or recurrent diarrhea, mucous purulent and bloody stool with abdominal pain, and varying degrees of systemic symptoms (Ng et al., 2017; Wu et al., 2018). The pathogenesis and exact etiology of the disease has not been clearly explained so far. Current research suggests that the pathogenesis of UC is related to factors such as genetic susceptibility, intestinal mucosal barrier defects, dysregulated immune responses, intestinal microflora dysbiosis, and the external environment (Ungaro et al., 2017; Ramos and Papadakis 2019). Epidemiological studies showed that the incidence and prevalence of UC in western countries were relatively high, but the overall trend was stable (Ungaro et al., 2017). In the past 20 years, the incidence and prevalence of UC in Eastern countries have increased rapidly, and UC has gradually become a global disease (Hindryckx et al., 2016; Kamm 2017). Numerous studies (Farraye et al., 2010; Conrad et al., 2014; Choi et al., 2017)have shown that the longer the onset of ulcerative colitis, the higher the prevalence of colon cancer. The probability of developing colon cancer is 1.6% in 10 years, 8.3% in 20 years, and 18.4% in 30 years. Because of its complicated etiology, difficult cure, easy recurrence, poor prognosis, and increased risk of colon cancer, it has been listed as one of the modern refractory diseases by the World Health Organization (Ungaro et al., 2017). At present, the therapeutic drugs for UC mainly include anti-inflammatory drugs (5-aminosalicylic acid and glucocorticoids), immunosuppressants, and biological agents (Laharie 2017). With the in-depth study of the pathogenesis of UC, the treatment of UC has achieved certain results, but many patients are prone to recurrent attacks after drug withdrawal, which is difficult to cure. In addition, long-term use of conventional therapeutic drugs such as anti-inflammatory drugs, hormones, and immunosuppressants has great side effects (Troncone and Monteleone 2017; Chhibba and Ma 2020). Therefore, it is urgent to find new therapeutic targets to reduce the use of anti-inflammatory drugs, hormones, and immunosuppressants, and reduce adverse drug reactions. Clinical studies have shown that traditional Chinese medicine (TCM) has good clinical efficacy in treating UC, it can effectively improve clinical symptoms and quality of life, alleviate intestinal inflammatory response and the disease progress, and decrease the disease recurrence (Gong et al., 2012; Dai et al., 2017; Zheng et al., 2020; Shen et al., 2021; Hou et al., 2022; Yin et al., 2022).

Macrophages are the innate immune cells of the body, which play an irreplaceable role in humoral immunity and cellular immunity. Intestinal macrophages can mediate the inflammatory response, and their excessive activation will cause an imbalance in the regulation of inflammation and transform physiological inflammation into pathological intestinal injury. Studies found that compared with the lamina propria of normal mucosa, the number of intestinal mucosal macrophages in the local colonic tissue of patients with active UC was significantly increased and tended to be activated, suggesting that intestinal macrophages were involved in the occurrence and development of UC (Na et al., 2019). Intestinal macrophages are mainly derived from bone marrow monocytes, and macrophages are polarized into different subtypes according to the local microenvironment of the intestine. More and more studies have found that UC patients have abnormal intestinal macrophage polarization, suggesting that intestinal macrophage polarization may play a central role in the occurrence and development of UC. Restoring the balance between the two subtypes of intestinal macrophages may become an important means of clinical treatment of UC. In this review, we firstly summarized the clinical evidence of TCM in the treatment of UC. Secondly, we discussed the basic biology of macrophages, macrophage polarization, phenotype, and the important role of macrophage polarization in the pathophysiology of UC. Finally, we discussed the evidence of TCM alleviates UC by regulating macrophage polarization and the potential mechanism of TCM regulating macrophage polarization in the treatment of UC.

## 2 TCM for UC treatment

### 2.1 Understanding UC in TCM theory

According to the clinical manifestations of UC mucous purulent bloody stool and the characteristics of recurrent, persistent, and difficult to recover, UC belongs to the category of chronic dysentery (Zhang et al., 2017). In the theory of spleen and stomach, Li Dongyuan clarified that the disease is closely related to diet and emotion. Improper diet, invasion of external evil, internal injury of emotion, and so on are the main causes. The most common CM patterns of UC patients were large intestine dampness-heat, followed by spleen-kidney yang deficiency, liver depression and spleen deficiency, spleen-stomach weakness, and spleen deficiency with dampness accumulation, which reflected the progress of UC with different phases (Zhang X. et al., 2022). For UC patients with CM pattern of Large intestine dampness-heat, which clear heat and dampness, harmony qi and blood are needed; For UC patients with CM pattern of Spleen-kidney yang deficiency, which warm and tonify spleen and kidney are needed; For UC patients with CM pattern of liver depression and spleen deficiency, which sooth liver and strengthen spleen are needed; For UC patients with CM pattern of spleen-stomach weakness, which tonify the spleen and stomach are needed; For UC patients with CM pattern of Spleen deficiency with dampness accumulation, which tonify spleen and resolve dampness are needed (Zhang X. et al., 2022). Syndrome differentiation and treatment is one of the most important principles of TCM in the treatment of UC.

### 2.2 Clinical evidence of TCM for UC

In recent years, there are many clinical research reports on the treatment of UC with TCM. According to various clinical types, attack remission period, and syndrome types, a large number of clinical studies such as internal administration of TCM, enema, the combination of Chinese medicine and western medicine, comprehensive therapy, and so on have been carried out. A multicenter, randomized, and double-blind clinical trial of patients with moderately active and 5-aminosalicylic acid-refractory UC found that Qing-Chang-Hua-Shi granules could significantly improve clinical efficacy, clinical response, and mucosal response, and are efficacious in introducing clinical remission, mucosal healing, as well as in relieving mucus/blood stool (Shen et al., 2021). Another similar double-blind, double-dummy, multicenter, randomized, and controlled study including 320 active UC patients with TCM pattern of damp-heat accumulating in the interior showed that, compared with mesalamine enteric-coated tablet, Fufangkushen colon-coated capsule was similarly clinical remission and safe in the treatment of active UC (Gong et al., 2012). A systematic review and meta-analysis of randomized clinical trials found that Five-flavor Sophora falvescens enteric-coated capsule (FSEC) alone could significantly improve the clinical effective rate and effective rate of CM patterns (Hou et al., 2022). Two other similar systematic review and meta-analysis trials both recommended that adjuvant curcumin therapy with conventional western medicine (CWM) was significantly superior to WM treatment alone in the primary outcomes of clinical remission. Furthermore, compared with placebo, topical curcumin enema had advantages in clinical remission, clinical improvement, and endoscopic improvement (Zheng et al., 2020; Yin et al., 2022). For patients with mild to moderately active UC in the syndrome of spleen deficiency and dampness-heat, TCM could ameliorate bowel symptoms, and systemic symptoms and improve the total scores of the short form-36 health survey questionnaire and inflammatory bowel disease questionnaire including physical function, bodily pain, vitality, and mental health, and consequently improved the quality of life (Dai et al., 2017). In addition, compared with CWM treatment alone, TCM enema combined with CWM could significantly improve clinical efficacy, reduce the recurrence rate, decrease the level of the intestinal and systemic inflammatory response, promote the recovery of intestinal mucosal function, promote mucosal healing, and improve the quality of life of UC patients (Yan ZX. et al., 2021; Li Y. et al., 2021; Hao 2021; Zhang Q. et al., 2022). In a word, the available clinical data show that TCM is significantly beneficial for treating UC.

## 3 Macrophage

### 3.1 Basic biology of macrophage

A macrophage is a cellular component commonly found in various tissues in the body under normal physiological conditions (Gordon and Taylor 2005; Locati et al., 2020). Monocytes can be released into the circulatory system as macrophage precursors. Monocytes differentiate into macrophages or dendritic cells as they migrate from the circulatory system and exude the endothelium. Macrophages in most organs consist of a heterogeneous mixture of bone marrow-derived and embryonic progenitor-derived macrophages, whereas the resident macrophage population of the brain is derived only from the proliferation of embryonic progenitor cells (Viola and Boeckxstaens 2021). Macrophages residing in tissues can play the role of “sentinels”, regulating the dynamic balance of tissues by responding appropriately to physiological changes and external challenges. During these dynamic balance processes, tissues can recruit macrophages of different phenotypes from monocyte banks, such as blood, bone marrow, and spleen, or they can ensure a certain number of macrophages utilizing local proliferation (Geissmann et al., 2010; Jenkins et al., 2011). Numerous studies have shown that in inflammatory tissues, the differentiation of monocytes into macrophages coexists with the proliferation of macrophages resident in many tissues (Hashimoto et al., 2013; Epelman et al., 2014). Macrophages, an important component of innate immunity, are the first line of defense against infection (Lavin et al., 2015). Macrophages have a unique function to sense and respond to the invasion and tissue damage by infectious microorganisms through various scavenger receptors, pattern recognition receptors, and phagocytic receptors (Canton et al., 2013; Zagorska et al., 2014). Macrophages may also participate in homeostasis maintenance by using phagocytosis to remove dead cells and lipoproteins. When macrophages are dysfunctional, their ability to maintain homeostasis and sense signals of tissue damage is impaired, which may lead to inflammatory diseases.

### 3.2 Macrophage polarization and phenotype

**FIGURE 1:**
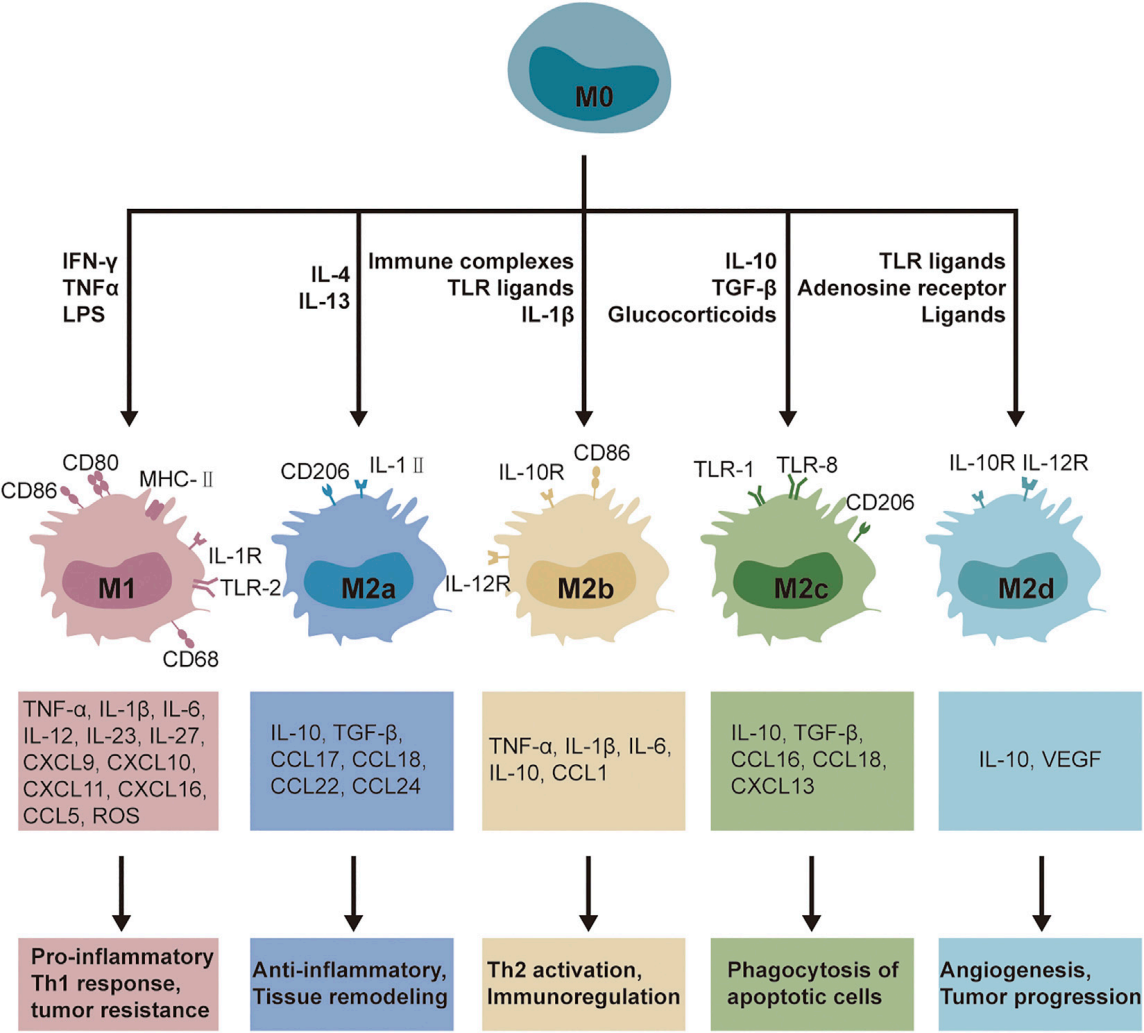
Overview of different biological and physiological features of macrophages polarization.

Macrophage polarization refers to the state in which macrophages are activated under certain space and time conditions. As a kind of highly plastic cell, the functional state and phenotype of macrophages are closely related to various signals in the tissue microenvironment. Therefore, the polarization state of macrophages is also changeable (Murray 2017). Macrophages are activated in a series of phenotypic forms in response to infection, damaged cells, activated lymphocytes, or other pathological conditions. Activated macrophages play different roles in the regulation of inflammation because of their different functional phenotypes, as is shown in Figure 1.

Activated macrophages can be broadly classified into classically activated macrophages (type M1) and alternatively activated macrophages (type M2) (Chambers et al., 2021). Th1-responsive activated macrophages are called M1 macrophages, i.e. classically activated, and are mainly activated by interferon-γ (IFNγ), lipopolysaccharide (LPS), and tumor necrosis factor-α (TNF-α). M1 macrophages are characterized by high expression of protein markers such as CD16/32, CD64, CD80, CD86, and so on, secretion of large amounts of pro-inflammatory cytokines such as interleukin-1β (IL-1β), IL-6, IL-12, IL-23, and TNF-α, chemokines such as CXC chemokine ligand 9 (CXCL9), CXCL10, CXCL11, and high expression of nitric oxide (NO), nitric oxide synthase (iNOS), and reactive oxygen species (ROS). These soluble mediators can further drive M1 polarization through positive feedback, enabling macrophages to display pro-inflammatory and anti-microbial activity (Yao Y. et al., 2019; Kang and Kumanogoh 2020). Functionally, M1 macrophages are significantly phagocytic, rapidly removing invading pathogens and mediating tissue damage, while promoting Th1 and Th17 cell-mediated immune responses and initiating inflammatory responses to protect the host, which play an important role in the early stages of inflammation. However, the excess can also impair tissue regeneration and wound healing, resulting in host damage (Shapouri-Moghaddam et al., 2018; Yunna et al., 2020).

Macrophages activated by Th2 response are called M2 macrophages, i.e., alternative activation, activated mainly by IL-4, IL-10, IL-13, TGF-β, and glucocorticoid. M2 macrophages are characterized by high expression of protein markers CD10, CD163, CD206, and arginase-1 (Arg-1), chitinase-like protein 3 (Chil3), and FIZZ1 (Wang LX. et al., 2019) and secrete large amounts of anti-inflammatory factors IL-4, IL-10, IL-13, transforming growth factor-β (TGF-β), Arg1, etc. In function, M2 macrophages exert anti-inflammatory activity and play a role in wound healing and fibrosis (Jetten et al., 2014; Murray et al., 2014; Sica et al., 2015). Based on the differences in activating molecules and gene expression profiles, M2 macrophages are further classified as M2a, M2b, M2c, and M2d macrophages. M2a macrophages, also known as wound healing macrophages, secrete anti-inflammatory factors, such as IL-10, TGFβ, and Arg1, induced by IL-4 and IL-13, which participate in the elimination of chronic inflammation and wound healing (Murray et al., 2014). M2b macrophages, also known as regulatory macrophages, highly express the pro-inflammatory cytokines IL-1β, IL-6, and TNF-α, while secreting a large amount of the anti-inflammatory cytokine IL-10 upon stimulation by immune complexes and TLR agonists (Yue et al., 2017). M2c macrophages, also known as acquired inactivated macrophages, are induced by IL-10 and high expression of CD206, IL-10, and TGF-β. In addition, IL-10 induces the further release of IL-10 and TGF-β by activating STAT3, which drives the conversion of macrophages to M2c type (Wynn and Vannella 2016). M2d macrophages, also known as tumor-associated macrophages, are co-stimulated by TLR ligands and A2 adenosine receptor agonists or induced by IL-6, highly express IL-10, TGF-β, and vascular endothelial growth factor (Shapouri-Moghaddam et al., 2018; Wang LX. et al., 2019).

During tissue repair, macrophages are in a dynamic state of transformation from the M1 phenotype to the M2 phenotype (Novak and Koh 2013; Chazaud 2014). In the early stage of inflammation, inflammatory mediators produced by M1 macrophages, such as TNF-α, IL-6, IL-1, and NO, stimulate innate immune cells (e.g., neutrophils) to resist pathogens invasion and protect the host (Shi and Pamer 2011; Sica et al., 2015). In the stage of receding inflammation, macrophages initiate the transition from the M1 phenotype to the M2 phenotype and gradually obtain the anti-inflammatory phenotype, which is characterized by down-regulation of inflammatory mediators, increased production of anti-inflammatory cytokines (e.g. TGF-β and IL-10), phagocytosis of apoptotic neutrophils and clearance of damaged cells (Daley et al., 2010; Gomez Perdiguero et al., 2015). During the proliferative phase, M2 macrophages produce a variety of growth factors, such as EGF, FGF, and VEGF, which induce the proliferation of various cells involved in the healing process and promote angiogenesis (Thomas et al., 2011; Jung et al., 2012). In the remodeling stage, macrophages promote the maturation of regenerative tissues, recombine the extracellular matrix, vascular system, and scar tissue, and promote the repair of damaged tissues (Mirza and Koh 2011; Willenborg et al., 2012).

### 3.3 Macrophage and intestinal mucosal healing

Achieving complete intestinal mucosal healing is the most desirable goal in the treatment of UC (Du et al., 2020). The healing of intestinal mucosa can not only significantly alleviate the disease process and improve clinical symptoms, but also significantly reduce the incidence of complications and prevent disease recurrence (Froslie et al., 2007; Pineton de Chambrun et al., 2010). Macrophages are effective targets for promoting intestinal mucosal regeneration in UC patients. When the intestinal epithelial barrier is damaged, macrophages are essential for the reconstruction of the intestinal epithelial barrier. In the process of intestinal mucosal repair, macrophages are indispensable participants in the repair process by directly promoting their proliferation and differentiation or creating a microenvironment conducive to epithelial regeneration and mucosal healing through close intercellular interactions with multiple intestinal epithelial cells (Na et al., 2019). Studies have shown that dextran sulfate sodium (DSS) can damage the intercellular junctions between colonic mucosal cells and cause colitis (Fan et al., 2020). However, macrophages accumulated in the intestinal tract of DSS-treated mice, and mice with macrophage-colony stimulating factor-1 (CSF-1) deficiency could not recruit monocytes after intestinal epithelial barrier disruption, so their colonic epithelial progenitor cells could not repair intestinal epithelial injury normally (Pull et al., 2005). Abnormal macrophages in UC patients affect the normal intestinal mucosal repair process.

### 3.4 Macrophage and T cell

Numerous experiments have demonstrated the important role of immune cells in the pathogenesis of IBD. For example, Th17 cells and their production of IL-17 are abundant in the intestinal mucous layer of IBD patients, and the latter leads to the damage of intestinal mucosa and aggravates the activity of the disease (Jiang et al., 2014). Th1 polarization is also associated with colonic inflammation, mainly through the induction of IFN-γ and TNF-α production (Yang et al., 2017). Among the immune cells, intestinal macrophages also play an important role. They are the main population of intestinal mucosal antigen-presenting cells, and determines the response type of T lymphocytes to intestinal antigens (Mahida 2000). As an antigen-presenting cell, macrophages play an important role in regulating innate and adaptive immune response and play an important role in UC tissue injury and promoting tissue repair (Gren and Grip 2016). After stimulation and activation, M1 macrophages secrete a large number of pro-inflammatory cytokines such as IL-1 β, IL-12, IL-23, TNF-α, etc., and participate in the phagocytosis of bacteria, chemotaxis of inflammatory cells, promotion of Th1 and Th17 cell-mediated immune response by high expression of inducible iNOS to decompose L-arginine to produce the NO and ROS, and exert host immune function, resulting in inflammatory damage of intestinal tissue in IBD patients. M2 macrophages can secrete immunosuppressive factors such as IL-10 and chemokines (CCL17, CCL22, CCL24) mediated by IL-4, IL-13 and TGF-β, which activate Th2 type immune response while inhibiting Th1 type immune response, play an anti-inflammatory role, and promote wound repair and fibrous degeneration.

### 3.5 Regulating the balance of M1/M2 macrophage polarization may be an effective therapeutic target in UC

Intestinal macrophages are closely related to the occurrence and development of UC (Na et al., 2019). Intestinal macrophages play an important role in the intestinal inflammatory microenvironment. They not only have the function of phagocytosis of pathogens, but also can secrete a variety of cytokines, act with a variety of immune cells, and participate in the occurrence, development, and persistence of UC. Compared with the macrophages in the lamina propria of normal mucosa, the number of intestinal mucosal macrophages in the local colonic tissue of active UC patients significantly increased and tended to be activated, suggesting that intestinal macrophages play an important role in the occurrence and development of UC.

Numerous studies have shown that regulating the polarization balance of macrophages is the top priority in the treatment of UC (Yao DB. et al., 2019). The pathogenesis of UC is the result of multiple factors, but its essence belongs to chronic inflammation. Inflammation is an indispensable means of defense against foreign bodies. Moderate inflammation is beneficial to the human body. If tissue integrity and body balance are restored in the process of repair, inflammation will disappear automatically. However, if inflammation is allowed to develop in an uncontrollable state, it will damage normal tissue function and eventually lead to organ failure (Wynn and Vannella 2016). Different polarization states of macrophages act as the “wind vane” of the development of inflammation. When macrophages toward an M1 phenotype, they will release excessive pro-inflammatory cytokines, and the inflammation becomes more and more serious. When macrophages toward the M2 phenotype, they will release anti-inflammatory factors, stop the process of inflammation, repair tissue damage and restore body balance. Therefore, it is of great significance for clinical prevention and treatment of UC to maintain the dynamic balance of M1/M2 by regulating the polarization of macrophages.

## 4 TCM alleviates UC by regulating macrophage polarization

According to TCM, the basic pathogenesis of UC is dampness and heat accumulating in intestines, qi stagnation and collateral stasis, mostly caused by exogenous seasonal pathogen, dietary irregularities (uncleanliness), internal injury of emotion, deficiency of spleen and kidney, phlegm, dampness, heat, toxin, blood stasis and deficiency affect each other and transform, which are accumulated in intestines, disorder of qi and blood, and then develop the disease (Shen et al., 2019). TCM is effective in the treatment of UC, which has unique advantages in promoting the recovery of intestinal mucosa, alleviating clinical symptoms and reducing recurrence. Intestinal mucosal healing is considered to be the core of the treatment of UC. In the process of intestinal mucosal repair, the polarized reconstruction of macrophages maintains the intestinal microenvironment homeostasis, which is a typical process of promoting mucosal inflammatory repair and a key link that cannot be ignored in initiating tissue regeneration (Na et al., 2019). The polarization of macrophages is intrinsically related to the theory of yin and yang in TCM. M1 and M2 macrophages in macrophage polarization play an important role in maintaining human immune homeostasis. The imbalance in the polarization process can lead to disease, as the saying goes, “predominance of yin leading to disorder of yang, predominance of yang leading to disorder of yin, predominance of yang generating heat, predominance of yin produces cold”. Therefore, the key to macrophage polarization regulation is to restore the balance of macrophage polarization, so that M1 and M2 macrophages can reach a balanced state of mutual restraint and mutual use. TCM treatment focuses on a holistic concept and syndrome differentiation and treatment, through adjusting the balance of yin and yang, strengthening vital qi and eliminate pathogenic factor, and treating the origin of the disease to achieve the purpose of treatment. Numerous modern studies have shown that TCM compound and extracts of single TCM can improve the intestinal inflammatory microenvironment by regulating macrophage polarization, thereby playing a therapeutic role in UC.

### 4.1 TCM compound alleviates UC by regulating macrophage polarization

In recent years, under the guidance of the theoretical system of TCM, researchers have actively explored the treatment of UC by adjusting the polarization of macrophages to improve the intestinal microenvironment with TCM compound. We collected and summarized the research in this area, as shown in Table 1. Baitouweng decoction is the most commonly used TCM formula in the clinical treatment of UC, followed by Shenling Baizhu San (Zhang X. et al., 2022). Baitouweng decoction is consist of *Pulsatilla chinensis* (Bunge) Regel., Radix, *Phellodendron chinense* C.K. Schneid., Cortex, *Coptis chinensis* Franch., Rhizome, and *Fraxinus rhynchophylla* Hence., Cortex, which has the effect of clearing heat and detoxification, cooling blood and stopping dysentery. It is regarded as a classic prescription for treating dysentery by doctors of all ages and is now widely used in the treatment of UC. Clinical studies showed that compared with the simple use of mesalazine, the combined medication of the modified baitouweng decoction and mesalazine improved the effective rate of UC and the optimal clinical outcome index ratio (Wang et al., 2018). A systematic review and meta-analysis of RCTs involving 842 UC patients showed that baitouweng decoction combined with mesalazine treatment had a better comprehensive efficacy, endoscopic mucosal efficacy, and the improvement effects on abdominal pain and bloody purulent stool than mesalazine treatment alone (Miu et al., 2018). Chen et al. (Xuan-Qing et al., 2021) reported that Baitouweng decoction could rescue body weight loss, reduce the DAI and histopathological scores, decrease the levels of IL-6, IL-1β, and TNF-α, and improve DSS-induced UC, which might be related to the inhibition of M1 macrophage polarization.

**TABLE 1:** TCM compound alleviates UC by regulating macrophage polarization.

TCM compound	Cellular or animal models	Main findings	Mechanism	Refer
Baitouweng decoction	DSS-induced colitis in mice	1) Improved colon shortening, reversed loss of body weight, decreased DAI scores	1) IL-1β↓, IL-6↓, TNF-α↓	Xuan-Qing et al. (2021)
		2) Ameliorated colonic pathological damage	2) Attenuated DSS-induced colitis by suppressing the activation of the IL-6/STAT3 signaling pathway	
			3)Inhibited M1 macrophage polarization	
Shenling Baizhu San	DSS-induced colitis in mice, TNFα-treated RAW 264.7 cell	1) Rescue colitis- caused epithelial deconstruction and weight loss	1) CXCL1↓, IL-17↓, ROS↓	Yu et al. (2022)
		2) Improved colonic pathological damage	2) Rescued TNFα-induced pyroptosis of intestinal organoids	
		—	3) Suppressed macrophage transition to pro-inflammatory phenotype (M1)	
		—	4) Decreased the recruitment of macrophage by epithelial cells	
Wumeiwan	DSS-induced colitis in mice	1) Improve inflammatory cell infiltration and crypt injury in the colonic tissues of colitis mice	1)IL-10↑, TGF-β↑, Arg1↑, TNF-α↓, IL-1↓, IL-1β↓, IL-6↓, IRF5↓	Yan et al. (2022)
		2) Decreased histopathological scores	2)Decrease macrophage infiltration and activation	
		3) Decreased DAI scores and alleviated colon shortening	3)Inhibited M1 macrophage polarization and promoted M2 macrophage polarization	
Gegen Qinlian decoction	DSS-induced colitis in mice	1) Ameliorated colon shortening, reversed loss of body weight, decreased DAI scores	1) IL-10↑, IL-1β↓, IL-6↓, TNF-α↓	Xu (2021)
		2) Improved colon tissue injury	2) Decrease the percentage of M1 macrophages and increase the percentage of M2 macrophages	
		3) Alleviated the histopathologic damage		
Banxia Xiexin decoction	TNBS-induced colitis in rats	1) Decreased DAI scores and colon weight index	1) IL-4↑, IL-10↑, SOD↑, 8-Oxoguanine↑, Nrf2↑, IL-1β↓, IL-6↓, TNF-α↓, IL-17↓, IL-23↓, COX-2↓, p-p65↓, MDA↓, MPO↓, 8-Oxoguanine↓	Wang et al. (2021a)
		2) Alleviated the histopathologic damage	2) Possessed the potential of anti-inflammation and anti-oxidation	Chen et al. (2015a)
Guchangzhixie capsule	DSS-induced colitis in mice	1)Relieved the epithelial structural collapse	1) CXCL1↓, IL-17↓, ROS↓	Yan et al. (2021a)
		2)Alleviated the severity of fecal blood and albumin, shortened colon length	2) Restored bacterial diversity of UC mice	
		—	3) Suppressed macrophage transition to proinflammatory phenotype	
Xian-He-Cao-Chang-Yan formula	DSS-induced colitis in mice, TNFα-treated RAW 264.7 cell	1) Decreased DAI scores, attenuated the colon shortening	1)Suppressed the M1 polarization of macrophages and increased the M2 polarization of macrophages	Li et al. (2021a)
		2) Alleviated the histopathologic damage	2)Regulated energy metabolism in macrophages through AMPK	
		3) Reduced the levels of HK2 and lactic acid	—	

Shenling Baizhu San (SBS) is consisting of ten herbs including *Panax ginseng* C. A. Mey, *Amomum aurantiacum* H. T. Tsai Et S. W. Zhao, *Lablab semen album*, *Poria cocos* (Schw.) Wolf, *Rhizoma dioscoreae*, *Coicis semen*, *Atractylodes macrocephala* Koidz, *Nelumbinis plumula*, *Platycodon grandiforus*, and *Licorice*, which is a classic prescription for replenishing Qi to invigorate the spleen, nouvish the stomach, and remove dampness infiltration wet. A great many clinical studies have confirmed that SBS is effective in the treatment of UC. A meta-analysis of RCTs involving 1177 UC patients showed that the therapeutic efficacy of SBS combined with aminosalicylic acid preparation was better than aminosalicylic acid preparation alone in the treatment of UC, and could significantly reduce the DAI scores and the levels of serum cytokines (Weng et al., 2017). Another systematic review and meta-analysis of RCTs involving 1,489 cases showed that compared with the western medicine group, SBS combined with the western medicine group could improve the combined risk ratio of clinical efficacy, and the DAI scores, and decrease the inflammatory factors levels of IL-17, IL-23, and TNF-α(Wen et al., 2017). In rats with ulcerative colitis, SBS could upregulate the expression of IκBα, downregulate the protein and mRNA expression of NF-κB p65, IκKβ, reduce the levels of serum TNF-α, IL-6, and IL-1β, which suggested that SBS suppressed inflammatory response and alleviated colonic tissue injure by inhibiting macrophage transition to M1 phenotype (Li ZH. et al., 2020). In addition, Yu et al. (Yu et al., 2022) reported that SBS suppressed experimental colitis progression, reduced inflammatory injury, and alleviated mucosal healing via inhibiting the polarization of macrophages to the M1 phenotype and reducing decreased the recruitment of macrophages by epithelial cells.

Wumeiwan originates from the“Treatise on Cold Damage Diseases” written by Zhang Zhongjing and has been widely used to treat UC. Wumeiwan is consisting of *Coptis chinensis*, *Cinnamomum cassia*, *Smoked plum*, *Zanthoxylum*, *Asarum sieboldii*, *Aconitum carmichaelii*, *Phellodendron amurense*, *Panax ginseng*, *Angelica Sinensis*, and *Zingiber officinale*. Clinical studies have shown that Wumeiwan and its modified formula have a significant and definite effect in the treatment of UC. A systematic review and meta-analysis including 11 RCTs showed that compared with CWM treatment alone, Wumeiwan combined with CWM treatment had a better clinical efficacy and endoscopic improvement (Chen XL. et al., 2015). Another similar systematic review and meta-analysis of RCTs including 2917 UC patients showed consistent results that integrated Wumeiwan and CWM therapy was more effective than CWM treatment alone in the indicators of clinical efficacy and endoscopic improvement. In addition, The use of Wumeiwan could reduce the incidence of adverse reactions (Wu N. et al., 2021). In 2,4,6-trinitrobenzenesulfonic acid (TNBS) induced ulcerative colitis rats, Wumeiwan could alleviate colonic tissue damage and improve disease activity index (DAI), the mechanisms might be related to reducing the levels of myeloperoxidase (MPO) activity, IL-6, and TNF-α, and decrease the expression of cyclooxygenase-2 (COX-2) protein (Ma et al., 2021). Du et al. (Du et al., 2021) reported that Wumeiwan could reduce DAI and histopathological scores in the UC rats by inhibiting the inflammatory reaction. In DSS-induced acute colitis mice, Wumeiwan could increase the percentage of M2 macrophages and decrease the percentage of M1 macrophages, and significantly reduce the secretion of pro-inflammatory factors, including TNF-α, IL-1β, and IL-6, by inhibiting M1 macrophage polarization and increase the secretion of anti-inflammatory factors, including IL-10, Arg-1, and TGF-β1, in colon tissues by promoting M1 macrophage polarization (Yan et al., 2022).

Gegen Qinlian decoction, a classic TCM compound, encompasses *Radix Puerariae*, *Scutellariae*, *Coptidis Rhizom*a, and *Licorice*. Recent research showed that Gegen Qinlian decoction combined with CWM seemed to be more effective in UC than CWM treatment alone (Huang et al., 2022). A systematic review of randomized controlled trials including 2028 UC patients showed consistent results that Gegen Qinlian decoction treatment alone or Gegen Qinlian decoction combined with CWM treatment had potential benefits in curing UC (Fan Y. et al., 2019). A large number of studies have shown that Gegen Qinlian decoction can exert the therapeutic effects against UC by regulating immune response, protecting intestinal mucosal barrier, anti-oxidative stress, inhibiting inflammatory response, and regulating intestinal flora (Zhao et al., 2020; Li Q. et al., 2021; Wang Y. et al., 2021; Liu et al., 2021; Zhao et al., 2021). In DSS-induced acute colitis mice, Gegen Qinlian decoction could improve the weight loss, colon shortening, reduce the DAI scores, and alleviate colonic pathological damage, by inhibiting M1 macrophage polarization, promoting M2 macrophage polarization, thereby down-regulating the expression levels of pro-inflammatory factors including IL-6, TNF-α, and IL-1β, and up-regulating the expression of anti-inflammatory factors including IL-10 (Xu 2021).

Banxia Xiexin decoction, an ancient TCM compound prescription composed of seven Chinese herbs, including *Pinelliae Rhizoma*, *Zingiberis Rhizoma*, *Scutellariae Radix*, *Glycyrrhizae Radix*, *Jujubae Fructus*, and *Coptidis Rhizoma*, has been widely used in gastrointestinal diseases such as UC. Clinical observation and meta-analysis showed that Banxia Xiexin decoction and its modified formula were safe and effective in the treatment of UC (Zhu JB. et al., 2016; Chi 2022). In TNBS-induced acute UC rats, Wang et al. (Wang W. et al., 2021) reported that Banxia Xiexin decoction could relieve the severity of UC to different degrees, including decreased the DAI scores, relieved the colonic congestion and edema, and improved colonic pathological damage. In addition, studies found that Banxia Xiexin decoction could significantly decrease the levels of anti-inflammatory cytokines, including IL-1β, TNF-α, IL-17, and IL-23, and significantly increased the levels of pro-inflammatory cytokines, including IL-4, IL-10, suggesting Banxia Xiexin decoction might improve the severity of UC by promoting M1 macrophage polarization, inhibiting M2 macrophage polarization, and restoring the balance of pro-inflammatory/anti-inflammatory axi (Chen G. et al., 2015; Wang W. et al., 2021).

The Guchangzhixie capsule is composed of *Mume Fructus*, *Corydalis rhizome*, *Papaveris pericarpium*, *Zingiberis rhizoma*, *Coptidis rhizoma*, and *Aucklandiae radix*, which has been widely utilized against UC. In a murine model of experimental colitis, Guchangzhixie capsule could inhibit experimental colitis progression including alleviating the severity of fecal blood and albumin, shortening colon length, relieving the epithelial structural collapse, and reversing the levels of IL-17 and CXCL1 in mouse serum by hampering macrophage infiltration and suppressing its transition to M1 macrophage polarization (Yan J. et al., 2021). Xian-He-Cao-Chang-Yan formula is composed of *Platycodonis Radix*, *Agrimoniae Herba*, *Cicadae Periostracum*, *Acori Tatarinowii Rhizoma*, *Aucklandiae Radix*, and *Coptidis Rhizoma*, which attenuated the increase of DAI score and the colon shortening, reduced the levels of inflammation in colon tissues and improved DSS-induced acute colitis in mice by suppressing the M1 polarization of macrophages and promoting the M2 polarization of macrophages (Li J. et al., 2021).

### 4.2 Extracts of single TCM alleviates UC by regulating macrophage polarization

In addition to TCM compound, many Chinese herbal and their active components have been proven to be able to treat UC by regulating the polarization state of macrophages. Table 2 lists some of extracts of single TCM that can ameliorate body weight loss and shortening of the colon length, decrease cumulative DAI scores, inhibit the infiltration of inflammatory cells, improve colonic pathological damage, and reduce MPO activity via regulation of macrophage polarization.

**TABLE 2:** TCM ingredient alleviates UC by regulating macrophage polarization.

TCM ingredient	Source	Cellular or animal models	Main findings	Mechanism	Refer
Acacetin	Acacia honey and citrus fruits	DSS-induced colitis in mice, LPS-treated RAW 264.7 cells	1) Ameliorated body weight loss, diarrhea, colon shortening	1) IL-1β↓, IL-6↓, TNF-α↓, NO↓, iNOS↓, COX-2↓	Ren et al. (2021)
			2) Alleviated the loss of mucosal architecture, ulcerations, and inflammatory infiltration	2) Attenuated DSS-induced colitis in mice by inhibiting inflammation and regulating the intestinal microbiota	
			3) Decreased macrophage infiltration and the levels of inflammatory mediators	—	
Asperuloside	*Hedyotis diffusa*	DSS-induced colitis in mice, LPS-treated RAW 264.7 cells	1) Improved weight loss, shortening of the colon length, and colon thickness, decreased DAI scores	1)IL-10↑, SOD ↑, GSH-Px↑, Nrf2↑, HO-1↑,NQO-1↑, MDA↓, TNF-α↓, IL-6↓, p65↓, ROS↓	Chen et al. (2021b)
			2) Remarkably decreased the MPO activity	2) Improved DSS-induced chronic colitis by alleviating inflammation and oxidative stress	
			3) Ameliorated colonic pathological damage	—	
Astragaloside IV	*Ligusticum chuanxiong*	DSS-induced colitis in mice	1) Alleviated body weight loss and the reduction in colon length, decreased cumulative DAI scores	1) IL-10↑, TGF-β↑, p-STAT3↑, TNF-α↓, IL-1β↓, iNOS↓, p-STAT1↓	Tian et al. (2021)
			2) Reduced MPO activity	2) Suppressed pro-inflammatory macrophage subsets and promoted pro-resolving macrophage subsets	
			3) Decreased histopathological scores	3) Modulated macrophage polarization by remodeling the STAT signaling	
Berberine	*Coptidis rhizoma, Phellodendri chinensis cortex*	DSS-induced colitis in mice	1) Ameliorated DSS-induced colon shortening	1) AKT1↑, SOCS1↑, p-p65↓, IL-1β↓, IL-6↓, IL-12↓, TNF-α↓, iNOS↓, NO↓	Liu et al. (2018)
			2) Improved colon tissue injury	2) Berberine inhibits macrophage M1 polarization via AKT1/SOCS1/NF-κB signaling pathway	
			3) Significant reduction in the DAI scores	—	
Berberine	*Coptidis rhizoma, Phellodendri chinensis cortex*	DSS-induced colitis in mice	1) Alleviated body weight loss	1) IL-10↑, TGF-β↑, Arg1↑, TNF-α↓, IL-6↓, iNOS↓, p50↓, p65↓	Xiong et al. (2021)
			2) Improved colonic pathological damage	2) Inhibited M1 macrophages polarization, promoted M2 macrophages polarization, improved the intestinal inflammatory environment, and relieved colitis by inhibiting NF-κB signaling pathway	
Curcumin	*Curcuma longa*	DSS-induced colitis in mice	1) Improved the body weight, colon weight and length, colonic weight index	1) IL-33↑, IL-10↑, IL-1β↓, IL-6↓, CCL-2↓, TLR2↓, TLR4↓, MyD88↓, NF-κBp65↓, p38MAPK↓, AP-1↓	Kang et al. (2021b)
			2) Significantly downregulated the pathological damage scores	2) Exerted therapeutic effects in colitis mice by regulating the balance of M1/M2 macrophage polarization and TLRs signaling pathway	
Polysaccharide from Dictyophora indusiata	*Dictyophora indusiata*	DSS-induced colitis in mice	1) Reduced DAI scores and MPO activity, relieved splenomegaly	1) GSH↑, HO-1↑, Bcl2↑, MDA↓, TNF-α↓, IL-1β↓, IL-6↓, IL-18↓, NLRP3↓, p-STAT3↓, p-IκBα↓	Wang et al. (2019b)
			2) Alleviated the histopathologic damage, including ameliorated disrupted tissue architecture, inflammatory infiltration, hyperemia and the disappearance of intestinal crypts and goblet cells	2) Inhibited DSS-induced apoptosis and deletion of tight junction protein in colonic tissues	
			—	3) Alleviated DSS-induced colitis via regulating macrophage subset	
Didymin	Fruits of rue family, such as oranges, lemons, and bergamot	DSS-induced colitis in mice	1) Remarkedly decreased the DAI scores, alleviated colon shortening	1) Arg1↑, IL-10↑, Chil3↑, Retnla↑, TNF-α↓, IL-1↓, IL-6↓, Nos2↓	Lv et al. (2021)
			2) Inhibited MPO activity, reduced neutrophils infiltration and improved histological damage	2) Decreased the proportion of M1 macrophages and comparatively increased the number of macrophages, and induced M1- to M2-like macrophage conversion	
Dioscin	*Dioscorea nipponica Makino*	DSS-induced colitis in mice, INF-γ/LPS-treated RAW 264.7 cells	1) Reversed loss of body weight, decreased DAI scores, extended colon length	1) IL-10↑, TNF-α↓, IFN-γ↓, and IL-6↓, iNOS↓, IL-1β↓, NO↓	Xie et al. (2021)
			2) Inhibited the infiltration of inflammatory cells, and relieved damage to the colonic mucosa	2) Reduced M1 macrophage polarization and facilitated transition of RAW264.7 cells towards M2 Phenotype	
			—	3) Protected mice against DSS-induced UC by regulating mTOR signaling, thereby adjusting macrophage metabolism and polarization	
Gentiopicroside	*Gentiane Radix Et Rhizoma*	DSS-induced colitis in mice	1) Increased the body weight and colon length, decreased colon weight and colonic weight index	1) IL-4↑, IL-10↑, CD206↑, IL-6↓, IL-12↓, iNOS↓, p-JAK2/JAK2↓, p-STAT3/STAT↓	Wu et al. (2021a)
			2) Decresed DAI scores	2) Down-regulated the phosphorylation of the JAK2/STAT3 pathway	
			3) Improved histopathological damage	3) Regulated the polarization of colonic macrophages towards M2 macrophages	
Ginsenoside Rg1	*Panax ginseng*	DSS-induced colitis in mice	1)Improved mouse body weight, mouse final weight, colonic weight, colonic length, colonic weight index	1) IL-4↑, IL-10↑, Arg1↑, IL-6↓, IL-33↓, CCL-2↓, TNF-α↓, Rock1↓, RhoA↓, Nogo-B↓, MIF-1↓, PIM-1	Long et al. (2022)
			2) Ameliorated pathological damage	2) Ameliorated colitis by regulating M1/M2macrophage polarization and microbiota composition	
			3) Improved the diversity of colonic microbiota	3) Inhibited the activation of Nogo-B/RhoA signalling pathway	
Loganin	*Cornus officinalis*	DSS-induced colitis in mice	1) Decreased body weight loss, colon shortening, and DAI scores	1) IL-1β↓, IL-6↓, TNF-α↓, MCP-1↓, CXCL10↓, COX-2↓	Liu et al. (2020)
			2) Reduced myeloperoxidase (MPO) activity and ameliorated pathologic abnormalities	2) Remarkably reduced macrophage M1 polarization	
			—	3) Inhibited M1 macrophage-mediated inflammation	
Lupeol	Vegetables, fruits, medicinal plants	DSS-induced colitis in mice	1) Mitigated intestinal inflammation and improved survival from lethal colitis	1) IL-10↑, IGF-1↑, Arg-1↑, CD206↑, IL-12↓, IL6↓, IL-1β↓, TNFα↓, iNOS↓, CD86↓, p38↓	Zhu et al. (2016a)
			2) Reduced histological score	2) Ameliorated mitigated intestinal inflammation through inhibiting M1 and promoting M2 macrophages	
Platycodin D	*Platycodonis Radix*	DSS-induced colitis in mice	1) Improved the weight loss, colon shortening, and reduced DAI scores	1) IL-10↑, Arg1↑, TNF-α↓, IL-6↓, IL-1β↓, iNOS↓	Guo et al. (2021)
			2) Alleviated colonic pathological damage	2) Inhibited the M1 macrophage polarization and promoted the M2 macrophage polarization	
			3) Inhibited the levels of inflammation in peripheral and colon tissues	3)Protected intestinal barrier function and improved intestinal inflammation by regulating macrophage polarization via activating AMPK	
Punicalagin	*Pomegranate*	LPS-induced RAW264.7 cells	1) Reduced LPS-Induced NO and PGE2 production	1) IL-1β↓, IL-6↓, TNF-α↓ TLR4↓, phosphorylation of IκBα↓	Xu et al. (2014)
			2) Inhibited LPS-Induced iNOS and COX-2 expression	2) Inhibited LPS-induced inflammation via the suppression of TLR4-Mediated MAPKs and NF-κB Activation	
Rosmarinic acid	*Prunella vulgaris*	DSS-induced colitis in mice, INF-γ/LPS-treated RAW 264.7 cells	1) Improved the weight loss, diarrhea, and hematochezia	1) Arg1↑, Mrc2↑, Dectin-1↑, TNF-α↓, IL-1β↓, NOS2↓, CCL4↓, IL-12↓	Mai et al. (2020)
			2) Alleviated colonic pathological damage	2) Improved UC by regulating macrophage polarity	
			—	3) Restricted macrophage polarization toward M1 depending on the inhibition of NF-κB p65 nuclear translocation by increasing HO-1	
Safranal	*Crocus sativus*	DSS-induced colitis in mice, LPS-treated RAW 264.7 cells	1) Alleviated the clinical symptoms, decreased DAI scores	1) IL-6↓, TNF-α↓, NO↓, iNOS↓, COX-2↓	Lertnimitphun et al. (2019)
			2) Decreased the severity of colon tissues inflammation, depth of inflammatory involvement, and crypt damage	2) Inhibited the phosphorylation of proteins ERK, JNK, and p38	
			3) Reduced the amount of macrophages accumulated in colonic tissue, mesenteric lymph nodes and spleens	3) Suppressed the phosphorylation and nuclear translocation of components of the MAPK and NF-κB pathways	
Tiliroside	Several edible plants or specific plant parts (fruits, leaves, or roots)	DSS-induced colitis in mice	1)Increased survival rate, decreased DAI scores, longer colon length	1) Arg1↑, Chil3↑, Cd206↑, IL-1β↓, iNOS↓, TNF-α↓	Zhuang et al. (2021)
			2)Reduced MPO activity	2) Promoted M2 macrophage polarization and inhibited M1 macrophage polarization	
			3)Ameliorated pathological changes of colons	3) Regulated macrophage polarization via blocking glycolysis pathway	
Toosendanin	*Melia toosendan Sieb et Zucc*	DSS-induced colitis in mice	1) Alleviated body weight loss, reduced DAI scores and the spleen weight	1) SOD↑, GSH↑, TNF-α↓, IL-1β↓, IL-6↓, MDA↓	Fan et al. (2019a)
			2) Reduced colonic tissue damage and tremendously increased MPO activity	2) Decreased the ratio of M1 macrophages and promoted that of M2 macrophages	
				3) Suppressed the activation of NLRP3 inflammasome and promoted the Nrf2/HO-1 signaling	
Total flavonoids of *Hedyotis diffusa Willd*	*Hedyotis diffusa Willd*	LPS-induced RAW264.7 cells	1) Inhibited the LPS-induced inflammatory response	1) IL-1β↓, IL-6↓, TNF-α↓, iNOS↓, NO↓	Chen et al. (2016)
				2) Exerted anti-inflammatory activity via suppression of the NF-κB and MAPK signaling pathways	
Vitexin	Several traditional Chinese medical herbs	AOM/DSS-induced colorectal cancer in mice	1) Attenuated colitis signs, colonic injury	1) IL-10↑, IL-1β↓, IL-6↓, TNF-α↓	Chen et al. (2021a)
				2) Decreased M1 phenotype macrophages in the adjacent noncancerous tissue	

As an isoquinoline alkaloid, Berberine (BBR) is one of the most thoroughly studied active ingredients. In the history of traditional medicine, TCM containing berberine has been used for more than 2,20 years (Imenshahidi and Hosseinzadeh 2016). Modern pharmacological studies have confirmed that BBR has anti-inflammatory, antioxidant, immunomodulatory, and anti-tumor effects (Abd El-Wahab et al., 2013). A large number of studies have shown that berberine can play a role in the treatment of UC by protecting intestinal barrier function, and regulating immune response and antioxidant stress (Habtemariam 2016). In DSS-induced colitis models, BBR has been shown to ameliorate the disease severity, alleviate colon shortening and colon histological damage, decrease the score of DAI, attenuate immune cell over-activation and inflammatory infiltrations, improve intestinal barrier function and restore the mucosal barrier homeostasis of UC (Cui et al., 2018; Liu et al., 2018; Li H. et al., 2020; Zhai et al., 2020; Wang YF. et al., 2021; Xiong et al., 2021; Dong et al., 2022). BBR significantly decreased the levels of colonic and serum pro-inflammatory cytokines including IL-1β, IL-6, TNF-α, IFN-γ, and IL-17A, and increased the levels of colonic and serum anti-inflammatory cytokines, including IL-10, TGF-β (Liu et al., 2018; Li H. et al., 2020; Wang YF. et al., 2021; Xiong et al., 2021). Berberine exerts a significant effect on macrophage polarization. Liu et al. (Liu et al., 2018) reported that berberine suppressed M1 macrophage polarization to protect against DSS-induced colitis via AKT1/SOCS1/NF-κB signaling pathway. Xiong et al. (Xiong et al., 2021) reported that berberine inhibited M1 macrophage polarization, promoted M2 macrophage polarization, increased M2/M1ratio, improved the intestinal inflammatory environment, and relieved colitis by inhibiting the NF-κB signaling pathway.

Curcumin is a main active ingredient of the rhizome of Curcuma longa (turmeric), which has a wide range of pharmacological effects, such as anti-inflammatory, antioxidant, antiplatelet regulating blood lipid and immunity (Borra et al., 2014; Li et al., 2015; Saji et al., 2018). Available clinical evidence suggested that as an adjuvant treatment in ulcerative colitis, curcumin was proved to be effective for clinical remission, quality of life improvement, endoscopic remission, and endoscopic improvement (Grammatikopoulou et al., 2018; Coelho et al., 2020; Sadeghi et al., 2020; Zheng et al., 2020; Yin et al., 2022). Curcumin could effectively inhibit the occurrence and development of DSS-induced UC and alleviate the disease. In DSS-induced colitis models, curcumin significantly increased the body weight and colon length, decreased the colon weight, colon mass index, and DAI score, and effectively improved histopathological damage in colitis mice (Zhang et al., 2019; Kang et al., 2021b; Xiao et al., 2022). Additionally, curcumin significantly reduced the levels of proinflammatory cytokines, including TNF-α, IL-1β, IL-6, CCL-2, and IL-17A, and significantly increased the levels of anti-inflammatory cytokines, including IL-33 and IL-10 (Zhang et al., 2019; Kang et al., 2021b; Xiao et al., 2022). About M1/M2 Macrophage Polarization, Kang, et al. (Kang et al., 2021b) the research found that curcumin significantly downregulated the percentage of M1 macrophage CD11b^+^F4/80^+^iNOS^+^ in colitis mice and significantly upregulated the percentage of M2 macrophage CD11b^+^F4/80^+^CD206^+^ and CD11b^+^F4/80^+^CD163^+^ macrophages, suggesting curcumin interfering with the process of experimental colitis effectively by regulating the balance of M1/M2 macrophage polarization.

In DSS-induced colitis mice models, Acacetin (Ren et al., 2021) and Asperuloside (Chen YE. et al., 2021) could reduce the levels of IL-1β, IL-6, and TNF-α, and ameliorated colitis via inhibiting macrophage inflammatory response. Punicalagin (Xu et al., 2014), Total flavonoids of Hedyotis diffusa Willd (Chen et al., 2016), and Safranal (Lertnimitphun et al., 2019) could inhibit inflammatory responses via suppression of the nuclear factor kappa-B (NF-κB) and mitogen-activated protein kinases (MAPK)signaling pathways, suggesting they decreased the levels of IL-1β, IL-6, and TNF-α are closely related to the inhibition of M1 macrophage polarization. Many experimental studies have also confirmed that CHB can significantly reduce the levels of pro-inflammatory factors, including IL-1β, IL-6, TNF-α, and IL-12, and increased the levels of anti-inflammatory factors, including IL-4, IL-10, TGF-β, and Arg1, thereby alleviating the severity of UC, by inhibiting the polarization of M1 macrophages and/or promoting the polarization of M2 macrophages, and regulating the balance of M1/M2 macrophage polarization (Zhu Y. et al., 2016; Liu et al., 2018; Fan H. et al., 2019; Wang YL. et al., 2019; Lertnimitphun et al., 2019; Liu et al., 2020; Mai et al., 2020; Chen Y. et al., 2021; Wu MM. et al., 2021; Kang et al., 2021b; Guo et al., 2021; Lv et al., 2021; Tian et al., 2021; Xie et al., 2021; Xiong et al., 2021; Zhuang et al., 2021; Long et al., 2022).

## 5 The potential mechanisms of macrophage polarization regulated by TCM

### 5.1 Regulation of signaling pathways related to macrophage polarization

The polarization and functional regulation of macrophages are completed under the joint action of many factors, which involve the regulation of multiple signal pathway transduction and transcriptional networks. At present, the recognized NF-κB, Peroxisome proliferator-activated receptor-γ (PPAR-γ), Phosphatidylinositol 3-kinase/Protein kinase B (PI3K/Akt), MAPK, Janus kinase/signal transducer and activator of transcription (JAK/STAT), Wnt/β-catenin, and Adenosine monophosphate-activated protein kinase (AMPK) signaling pathways are the key signal pathways of macrophage polarization and jointly regulate the phenotypic transformation of macrophages. In the process of studying the mechanism of macrophage polarization, it is found that TCM acts on the above signal pathways through multiple targets to regulate the process of macrophage polarization and effectively control inflammation.

#### 5.1.1 NF-κB signaling pathway

NF-κB, a transcription factor that can regulate the transcription of multiple proinflammatory cytokines, has been found to play an important role in the polarization of M1 macrophages (Bashir et al., 2016; Taniguchi and Karin 2018). NF- κB1 protein composed of P65/P50 subunit is the main activating molecule in the classical pathway of NF- κB signal. Under normal circumstances, NF-κB in macrophages binds to IκB in the cytoplasm and cannot enter the nucleus. When stimulated by LPS pro-inflammatory cytokines such as TNF-α, IL-1β, and NF-κB signal pathway is abnormally activated, resulting in phosphorylation of IκB kinase (IKK) complex and activation, which leads to the inactivation of IκBα after phosphorylation, which can not inhibit NF-κB, thus causing NF-κB phosphorylation and activation. Eventually, NF-kappa B accumulates in the nucleus (Greten et al., 2007). The activation and translocation of NF- κB in the nucleus can interact with the regulatory factors in the NF-κB promoter and enhancer, resulting in the transcription of some M1-related inflammatory genes dependent on p65-p50 NF-κB (Pantano et al., 2006; Porta et al., 2015). Studies have shown that the level of NF- κB p65 is enhanced and activated in macrophages isolated from intestinal samples of patients with IBD (Atreya et al., 2008). In LPS-stimulated RAW264.7 cells, asperuloside significantly could downregulate the level of nuclear p65, restore the level of cytosolic p65, suppress the translocation of NF-κB/p65 into the nucleus, decrease the mRNA expression of TNF-α and IL-6 in a concentration-dependent manner. Thus, these results suggested that asperuloside could inhibit M1 macrophage polarization by inhibiting the NF-κB signaling pathway (Chen YE. et al., 2021). In DSS-induced colitis, Rosmarinic acid (Mai et al., 2020) could improve weight loss, diarrhea, hematochezia, and colonic pathological damage by suppressing M1 Macrophage polarization via the inhibition of NF-κB p65 nuclear translocation. Xiong et al. (Xiong et al., 2021) reported that berberine could inhibit M1 polarization and promote M2 polarization in colonic macrophages, improve the intestinal inflammatory environment, and relieve colitis by inhibiting the NF-κB signaling pathway. Yan et al. (Yan et al., 2022) study found that Wumeiwan could inhibit M1 macrophage polarization and promote M2 macrophage polarization by inhibiting the activation of the NF-κB signaling pathway, thereby inhibiting inflammatory responses and alleviating colonic tissue damage in DSS-induced colitis.

#### 5.1.2 PPAR-γ signaling pathway

PPAR-γ is a kind of nuclear transcription factor activated by ligands, which plays an important role in inflammatory regulation, cell differentiation, and immune regulation. In recent years, studies have confirmed that activated PPAR-γ can inhibit the release of inflammatory mediators by competitively inhibiting the activation of transcription factors such as NF-κB, AP-1, and STAT (Kundu et al., 2014). The activation of PPAR-γ is also a key link in the polarization of macrophages to M2 type, which plays a pivotal role in inhibiting the inflammatory response of macrophages (Wen et al., 2018; Wen et al., 2020). Luo et al. (Luo et al., 2017) found that the use of PPAR-γ agonists could increase the transcription and expression of PPAR-γ, increase the proportion of M2 macrophages and reduce the inflammatory response, while the use of PPAR-γ inhibitors showed the opposite results, indicating that the activation of PPAR-γ contributed to the polarization of M2 macrophages and inhibited the inflammatory response. PPAR-γ has a protective effect on UC, which is negatively correlated with the severity of colitis. Wu et al. (Wu MM. et al., 2021) reported that Dioscin alleviated colitis via the promotion of M2 macrophage polarization by promoting the mammalian target rapamycin complex 2 (mTORC2)/PPAR-γ signaling pathway.

#### 5.1.3 PI3K/Akt signaling pathway

PI3K/Akt signal pathway plays an important role in the regulation of inflammatory response and macrophage polarization. The activation of PI3K or Akt can inhibit the activation of M1 macrophages induced by LPS and promote the activation of M2 macrophages (Vergadi et al., 2017; Sharif et al., 2019). PI3K can inhibit the pro-inflammatory response of macrophages stimulated by TLR, such as the inflammatory response in the process of LPS-mediated TLR4 activation (Sharif et al., 2019). Akt is the most significant effect factor of PI3K, which can be subdivided into three forms: Akt1, Akt2, and Akt3, which have different functions (Zhou et al., 2014). Akt1 can promote the polarization of M2 macrophages and inhibit the polarization of M1 macrophages, while Akt2 has the opposite effect (Covarrubias et al., 2015; Vergadi et al., 2017). Akt1 deletion promotes macrophage polarization to the M1 phenotype, while Akt2 deletion promotes macrophage polarization to the M2 phenotype (Arranz et al., 2012). In LPS-Induced RAW 264.7 macrophages, Heterophyllin B decreased the levels of IL-1β and IL-6 expression by suppressing the PI3K/Akt Pathways, thereby exerting anti-inflammatory activity (Yang C. et al., 2018). Fucoxanthin significantly attenuated LPS-induced ROS accumulation and pro-inflammatory cytokine expression in macrophages by regulating PI3K/AKT pathway. Peng et al. (Peng et al., 2022) study found that Xiaojindan could decrease the expression of M1 macrophage markers, including decreasing the release of NO and IL-6 in a dose-dependent manner and suppressing the mRNA expression of IL-1β, iNOS, COX-2, and TNF-α, by targeting PI3K/Akt pathway.

#### 5.1.4 MAPK signaling pathway

MAPK is a key hub for the transduction of extracellular signals to the nucleus, with a total of 14 members, including three major members: p38 MAPK, extracellular signal-regulated kinase (ERK), and c-Jun N-terminal kinase (JNK) (Zhang et al., 2021). The activation of the MAPK signaling pathway is a typical three-stage enzyme-linked reaction: MAPK kinase kinase-MAPK kinase-MAPK. After MAPK activation, c-Fos and c-Jun, members of the transcription activator protein-1 (AP-1) family, are further activated. Among them, p38 and ERK activate c-Fos, and JNK activates c-Jun. The activation of c-Fos and c-Jun enter the nucleus and also initiates the transcription process, which promotes the synthesis and release of inflammatory mediators in M1 macrophages. MAPK signaling pathway is involved in many biological processes including inflammation. The phosphorylation of p38 in macrophages is an important signal to regulate the production of pro-inflammatory factors such as IL-1β, IL-6, and TNF-α (Arthur and Ley 2013). It has been reported that the MAPK signaling pathway also regulates the expression of iNOS and COX2 genes in activated macrophages (Ronco 2014). In addition, NF-κB and MAPK signaling pathways promote each other and have a close relationship in the inflammatory response, and jointly regulate the phenotypic changes of M1 macrophages. Cell experiments showed that LPS induced M1 overexpression of inflammatory mediators such as iNOS and NO by activating MAPK and NF-κB pathways (Zhang et al., 2021). Chen et al. (Chen et al., 2016) reported that total flavonoids of *Hedyotis diffusa Willd* (TFHDW) decreased the production and mRNA expression of NO and pro-inflammatory cytokines such as TNF-α, IL-6, and IL-1β, as well as inhibited p38, JNK, ERK1/2, and IκBα phosphorylation in LPS-Induced RAW264.7 cells, suggesting that TFHDW suppressed inflammatory responses by inhibiting M1 macrophage polarization via suppression of the MAPK and NF-κB signaling pathways. In LPS-Induced RAW264.7 cells, safranal (Lertnimitphun et al., 2019) reduced NO, IL-6, and TNF-α production and suppressed the expression of iNOS and COX-2 via inhibiting the phosphorylation of JNK, ERK, p38, and IκBα, suggesting that safranal suppressed macrophage-mediated inflammatory responses via inhibition of M1 macrophage polarization by inhibiting MAPK and NF-κB signaling pathways. In the colitis mice model, curcumin alleviated experimental colitis by decreasing the percentage of M1 macrophages and increasing the percentage of M2 macrophages via inhibition of the TLRs/NF-κB-MAPK signaling pathway (Kang et al., 2021a).

#### 5.1.5 JAK/STAT signaling pathway

JAK/STAT is an important signal transduction pathway in the body, especially in the inflammatory response and macrophage polarization. Yang et al. (Yang et al., 2021) reported that berberine (BBR) significantly reduced the M1-specific markers (TNF-α and IFN-γ), increased the M2-specific markers (TGF-β and IL-10), promoted M2 macrophage polarization, and suppressed M1 macrophages polarization by activates IL-4/STAT6 signaling pathway. The results of *in vivo* and *in vitro* experiments (Xu et al., 2021) showed that baicalin reduced the levels of IL-12, TNF-α, and iNOS, inhibited the expression levels of iNOS, CCL-5, and p-STAT1 proteins, and increased the levels of IL-4 and IL-10, improved the expression levels of Arg1, MRC1, p-JAK1, and p-STAT6 proteins, and promote M2 macrophage polarization by activating JAK1/STAT6 pathway. In DSS-induced colitis mice, Wumeiwan regulated macrophage M1/M2 polarization in colonic macrophages to ameliorate colitis by activating the STAT6 signaling pathway (Yan et al., 2022). Astragaloside IV alleviated clinical disease activity, reduced the levels of the pro-inflammatory cytokine, increased the levels of the anti-inflammatory cytokine, and reduced the percentages of macrophages by suppressing M1 macrophage polarization partially by remodeling the STAT1 signaling pathway (Tian et al., 2021). Gentiopicroside could improve DSS induced intestinal injury and intestinal inflammation in UC mice, and inhibit the development of UC, by inhibiting the phosphorylation level of the JAK2/STAT3 pathway, promoting the polarization of macrophages to M2 macrophages, thereby down-regulating the secretion of intestinal proinflammatory factors and up-regulating the level of anti-inflammatory factors (Xie et al., 2021).

#### 5.1.6 Wnt/β-catenin signaling pathway

The classical Wnt/β-catenin signaling pathway is essential for cell growth, development, migration, cycle progression, and genetic stability (Nusse and Clevers 2017; Chae and Bothwell 2018). Research showed that activating the Wnt/β-catenin signaling pathway could promote M1 macrophage polarization and inhibit M2 macrophage polarization (Malsin et al., 2019). Wnt/β-catenin signal abnormal transduction plays an important role in the malignant progression of immune-inflammatory diseases by regulating the phagocytosis of macrophages (Au et al., 2018; Yuan et al., 2020). Recent studies have reported that activation of the Wnt/β-catenin signal pathway can lead to M2 macrophage polarization, promote macrophage proliferation and infiltration, and aggravate the progression of fibrotic disease and cancer cell migration (Yang Y. et al., 2018; Feng et al., 2018). Gong et al. (Gong et al., 2020) reported Fractalkine overexpression suppressed LPS-induced M1 macrophage polarization by activating the Wnt/β-catenin signaling pathway. In the DSS-induced colitis model, Berberine could significantly relieve colitis symptoms, improve intestinal damage, and repair the integrity of the intestinal epithelial barrier by activating the Wnt/β-catenin pathway (Hong et al., 2021).

#### 5.1.7 AMPK signaling pathway

AMPK is an important cellular metabolic receptor, which plays a key role in the regulation of cellular energy metabolism (Hardie 2011). The polarization of macrophages needs energy consumption, and oxidative metabolism also affects the polarization of macrophages. It has been reported that the activation of AMPK is involved in the polarization of macrophages (Ye et al., 2020). After the activation of AMPK, it can inhibit inflammation by inhibiting glycolysis, promoting oxidative phosphorylation, and changing the anti-inflammatory phenotype of macrophages. In addition, activation of AMPK can reduce the degradation of IκB and increase the activation of Akt, thereby suppressing the inflammatory response, effectively inhibiting M1 macrophages, and driving them to anti-inflammatory M2 phenotypic polarization (Sag et al., 2008). Li et al. (Li J. et al., 2021) reported Xian-He-Cao-Chang-Yan formula could ameliorate DSS-induced acute colitis by inhibiting M1 macrophage polarization and promoting M2 macrophage polarization through the regulation of macrophage energy metabolism via enhancing the activation of AMPK. In a murine model of colitis, Platycodin D could ameliorate the clinical symptoms and colonic pathological damage, reduce the levels of inflammation, and protect intestinal integrity via suppression of M1 macrophage polarization and promotion of M2 macrophage polarization by promoting activation of PI3K/Akt signaling pathway and suppressing of activation of NF-κB signaling pathways, which was closely related to activation of AMPK(Guo et al., 2021).

### 5.2 TCM induces macrophage polarization by regulating gut microbiota

Gut microbiota, which is composed of a large number of bacteria living in the intestinal tract of the host, has a complex composition and a wide variety of bacteria, which can be divided into three types: symbiotic bacteria, conditional pathogenic bacteria, and pathogenic bacteria. Under normal circumstances, the gut microbiota is in dynamic balance (Yang et al., 2022). Once the gut microbiota is out of balance, it will lead to the increase of conditionally pathogenic flora or pathogenic bacteria to the degree of pathogenicity (Shen et al., 2018), thereby leading to the occurrence of intestinal diseases (Kuhn et al., 2014). Therefore, the balance of gut microbiota, as a natural barrier, can effectively prevent the invasion of foreign pathogens (Yu 2018). Gut microbiota plays an important role in the pathogenesis, development, and recovery of UC. Related studies have shown that the change of gut microbiota in patients with UC is closely related to the degree of inflammation (Dai et al., 2021).

Gut microbiota and its metabolites can affect macrophage polarization. Qiu et al. (Qin et al., 2021) reported that there is gut microbiota imbalance in colitis mice, especially Bacteroides is directly related to the abnormal activation of M1 macrophages. The transplantation of fecal flora in healthy mice can inhibit the abnormal activation of M1 macrophages in DSS colitis mice. Recent research found that Fusobacterium nucleatum was a species of intestinal commensal bacteria with potential pathogenicity, which aggravated the course of UC progression by promoting M1 macrophage polarization (Liu et al., 2019). In the DSS-induced ulcerative colitis model, *Fusobacterium nucleatum* could increase the loss of body weight and colon length, inflammatory cell infiltration, mucosal necrosis, and intestinal permeability by promoting pro-inflammatory M1 macrophage levels and reducing anti-inflammatory M2 macrophage levels (Liu et al., 2019). Short-chain fatty acids (SCFAs), a predominant kind of gut microbiota metabolites, were important substances to maintain intestinal homeostasis, mainly including Acetate, Butyrate, and Propionate, which are anti-inflammatory properties and are demonstrated to relate to inflammation in chronic stages of diverse metabolic diseases (Morrison and Preston 2016; Weitkunat et al., 2017; Hirschberg et al., 2019). SCFAs were closely correlated with M1 macrophage polarization and M2 macrophage polarization. Wang et al. (Wang et al., 2020)found that Propionate, Butyrate and Acetate could directly inhibit M1 macrophages polarization and promote M2 macrophage polarization, and inulin could ameliorate the inflammation via SCFAs-inducing suppression of M1 macrophage polarization and facilitation of M2 macrophage polarization. In methionine-and choline-deficient (MCD)-induced hepatic inflammation, SCFAs attenuated MCD diet-induced hepatic inflammation by reducing macrophage infiltration, indicating inhibit M1 macrophage polarization (Deng et al., 2020). Trimethylamine N-Oxide (TMAO) is also one of the important metabolites of intestinal flora. Studies have found that TMAO participates in the intestinal mucosal inflammatory response, is closely related to the abnormal function of the intestinal mucosal barrier in UC, and aggravate the course of UC progression. Shi et al. (Shi et al., 2021) reported that TMAO treatment promoted M2 macrophage polarization without a significant effect on M1 macrophage polarization. Lactic acid, as a metabolic substrate of host and intestinal microbiota, could reduce the number of macrophages and the mRNA expressions of the iNOS and IL-6 of the M1-like phenotype with LPS stimulation, and increased the number of the percentage of M2 macrophages, suggesting lactic acid-mediated the phagocytosis of the macrophages and the macrophage polarization state (Sun et al., 2021).

**FIGURE 2:**
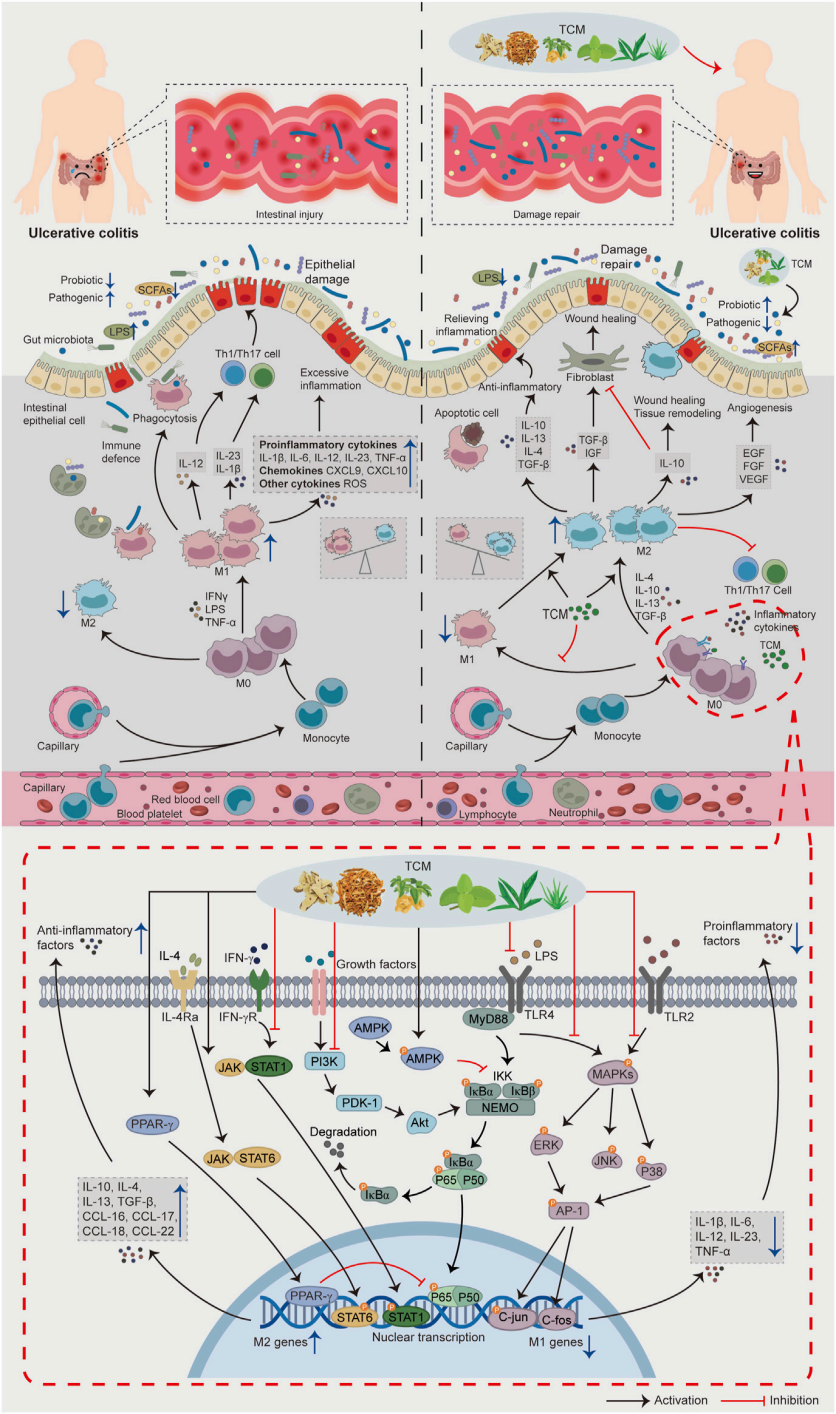
The role of intestinal macrophage polarization in the pathogenesis of UC and the potential mechanisms of TCM for UC based on intestinal macrophage polarization. When intestinal inflammation occurs in patients with UC, the lamina propria of the intestinal mucosa recruit a large number of monocytes and differentiate into immature M0 macrophages. Induced by a large number of pro-inflammatory factors (IFNγ, LPS, TNF-α), macrophages polarize toward the M1 phenotype and secrete a large number of pro-inflammatory cytokines, chemokines, and inflammatory mediators, causing an imbalance in the regulation of intestinal inflammation, transforming the physiological inflammatory response into pathological intestinal injury and aggravating the condition of UC. In addition, M1 macrophages can further aggravate the intestinal injury by secreting IL-12, IL-23, IL-1β and increasing Th1/Th17 activity. In the treatment stage, TCM can promote M2 macrophage polarization and inhibit M1 macrophage polarization by regulating the signaling pathways related to macrophage polarization, regulating gut microbiota and its metabolites, thus reducing the intestinal inflammatory response, promoting intestinal tissue repair, and alleviating UC progression.

Finally, according to the available fundamental evidence, a panorama of the role of intestinal macrophage polarization in the pathogenesis of UC and the potential mechanisms of TCM for UC based on intestinal macrophages polarization is drawn in Figure 2, hoping that intestinal macrophages polarization-based TCM strategies for UC treatment could be described and presented within a single framework.

## 6 Conclusions and prospects

Although a large number of review articles have been published on the treatment of UC with TCM, our work has something unique.1) As far as we know, this is the first relatively comprehensive review so far to explore the mechanism of TCM for UC based on intestinal macrophage polarization.2) According to the available fundamental evidence, this is the first comprehensive and systematic description and presentation of the role of macrophage polarization in the pathogenesis of UC and the way of TCM intervention from the whole, local to the cellular level.

As an immune-mediated chronic non-specific inflammatory disease, the occurrence of UC is related to the interaction of environmental, genetic, and immune factors, among which abnormal immune regulation is the crucial factor (Na et al., 2019). As an extremely abundant group of cells in the intestinal tract, intestinal macrophages not only have the function of phagocytosis of pathogens, but also secrete a variety of cytokines, interact with a variety of immune cells, and participate in the occurrence, development, and persistence of UC. The characteristics of intestinal macrophages are highly heterogeneous, pluripotency, and plasticity, under different local intestinal microenvironment stimulation, intestinal macrophages can be polarized into classical activated pro-inflammatory phenotype (M1) and alternative activated anti-inflammatory phenotype (M2). Its different polarization state is the “guide” to promoting the development and regression of intestinal inflammation. More and more studies have found that there is abnormal polarization of intestinal macrophages in patients with UC, which may play a core role in the occurrence and development of UC; restoring the balance between the two subtypes of intestinal macrophages may become an important means of clinical treatment of UC.

Intestinal macrophage polarization is a complex and continuous process. M1 macrophages and M2 macrophages can transform or coexist with each other. To clarify the relationship between the two macrophage phenotypes and UC, we need to analyze based on the overall microenvironment of the disease at that time. This holistic approach to analysis coincides with the holistic concept of TCM, thus linking the effects and related mechanisms of TCM for UC with macrophage polarization will enrich the scientific connotation of TCM for UC. Currently, many modern studies have shown that TCM can regulate intestinal macrophage polarization to improve the inflammatory microenvironment of the intestine and thus play a therapeutic role in UC. In regulating macrophage polarization, heat-clearing prescriptions have outstanding effects, such as Baitouweng decoction (Xuan-Qing et al., 2021), Gegen Qinlian decoction (Xu 2021), *Scutellariae Radix* (Zhu W. et al., 2016), *Coptidis Rhizoma* (Xiong et al., 2021), Prunella vulgaris (Mai et al., 2020) and so on, In addition, some prescriptions for activating blood and resolving stasis, such as *Curcumin* (Kang et al., 2021b), *Ligusticum chuanxiong* (Tian et al., 2021), and tonifying prescriptions, such as *Panax ginseng* (Long et al., 2022), Shenling Baizhu San (Yu et al., 2022) can also improve intestinal inflammation by regulating macrophage polarization in the treatment of UC.

Increasing evidence now supports that targeted therapy of macrophage polarization can re-establish intestinal immune microenvironment homeostasis and restore tissue homeostasis after inflammation, making macrophage polarization a new focus for exploring therapeutic approaches for UC. In recent years, although TCM research has paid more and more attention to the role of macrophage polarization in ulcerative colitis, few studies have been conducted to explore the mechanism of macrophage polarization regulation by TCM, and most of them are limited to the cellular level, and animal and clinical trials are scarce. In addition, most of the current studies have focused on the protection of tissue damage from a simple anti-inflammatory perspective or from a direct repair and regeneration perspective to achieve intestinal mucosal repair, with insufficient attention to the complete pathway chain to achieve tissue regeneration. Therefore, the clinical and basic research of TCM intervention in macrophage polarization should be further strengthened in the future to enrich the scientific connotation of TCM in the treatment of UC. In the future, we should pay attention to the treatment strategy of drugs based on the whole chain of inflammatory repair, so as to play the key role of immune cells related to tissue regeneration and repair, represented by M2 macrophages, in inflammatory diseases, and to highlight the science and effectiveness of the theory of TCM “strengthening vital qi and eliminate pathogenic factor”.

## Abbreviations

UC, Ulcerative colitis; TCM, traditional Chinese medicine; CWM, conventional western medicine; IFNγ, interferon-γ; LPS, lipopolysaccharide; TNF-α, tumor necrosis factor-α; Arg-1, arginase-1; DAI, disease activity index; MPO, myeloperoxidase; COX-2, cyclooxygenase-2; IL-1β, interleukin-1β; Nitric oxide (NO); iNOS, nitric oxide synthase; CXCL9, CXC chemokine ligand 9; CCL-5, chemokine-5; DSS, dextran sulfate sodium; TNBS, 2,4,6-trinitrobenzenesulfonic acid; CHM, Chinese herbal medicines; BBR, Berberine; NF-κB, nuclear factor kappa-B; MAPK, mitogen-activated protein kinases; PPAR-γ, Peroxisome proliferator-activated receptor-γ; PI3K/Akt, Phosphatidylinositol 3-kinase/Protein kinase B; AMPK, Adenosine monophosphate-activated protein kinase; JAK/STAT, Janus kinase/signal transducer and activator of transcription; ERK, extracellular signal-regulated kinase; JNK, c-Jun N-terminal kinase; AP-1, activator protein-1; SCFAs, Short-chain fatty acids; TMAO, Trimethylamine N-Oxide; MCD, methionine-and choline-deficient.
